# Co-Leadership – A Management Solution for Integrated Health and Social Care

**DOI:** 10.5334/ijic.2236

**Published:** 2016-05-23

**Authors:** Charlotte Klinga, Johan Hansson, Henna Hasson, Magna Andreen Sachs

**Affiliations:** Medical Management Centre (MMC), Department of Learning, Informatics, Management and Ethics, Karolinska Institutet, SE; Department of Social Work, Karolinska University Hospital, SE; Centre for Epidemiology and Community Medicine, Stockholm County Council, SE

**Keywords:** shared leadership, joint working, health care delivery, organizational sustainability

## Abstract

**Introduction:**

Co-leadership has been identified as one approach to meet the managerial challenges of integrated services, but research on the topic is limited. In the present study, co-leadership, practised by pairs of managers – each manager representing one of the two principal organizations in integrated health and social care services – was explored.

**Aim:**

To investigate co-leadership in integrated health and social care, identify essential preconditions in fulfilling the management assignment, its operationalization and impact on provision of sustainable integration of health and social care.

**Method:**

Interviews with eight managers exercising co-leadership were analysed using directed content analysis. Respondent validation was conducted through additional interviews with the same managers.

**Results:**

Key contextual preconditions were an organization-wide model supporting co-leadership and co-location of services. Perception of the management role as a collective activity, continuous communication and lack of prestige were essential personal and interpersonal preconditions. In daily practice, office sharing, being able to give and take and support each other contributed to provision of sustainable integration of health and social care.

**Conclusion and discussion:**

Co-leadership promoted robust management by providing broader competence, continuous learning and joint responsibility for services. Integrated health and social care services should consider employing co-leadership as a managerial solution to achieve sustainability.

## Introduction

Management of complex service innovations, as integrated health and social care organisations is known to be demanding. Unequal division of power between stakeholders and the following difficulties in balancing various interests have been identified as key challenges [1]. Might co-leadership be a possible management solution to address the challenges that arise due to organizational complexity? Health and social care often belong to separate organisational silos in welfare systems. In Sweden, as in most other countries, health and social care are governed by different jurisdictions and have different missions [2]. To deliver sustainable health and social care, cross-boundary collaboration is needed, although it may involve several challenges [3]. Coordination of health and social care through different forms of cooperation has been identified as a key element in reducing fragmentation of care and costs as well as improving quality of care and patient health outcomes [4,5,6]. In addition, collaboration between professionals from different sectors is likely to develop more people-centred and holistic care [7]. Individuals with complex health and social care needs, such as those with mental illness and disabilities, are particularly vulnerable to fragmented care. To decrease the fragmentation, a higher degree of cooperation between services or integration of services is required. This in turn increases the organizational complexity and thereby also the managerial challenges. Nonetheless, due to inherent differences between health and social services, difficulties in achieving efficient interaction prevail [8,9,10,11,12,13]. For example, separate funding streams, lack of economic incentives, poorly harmonized legal frameworks and different information and communication systems have created barriers [14]. Furthermore, differences in perceptions of responsibilities and of management and leadership role, as well as differences in organizational culture and resource availability have been identified as obstacles to cross-boundary interaction and collaboration [15,16,17].

Integrated organizational forms can involve collaboration between different disciplines in interprofessional teams, coordination of health and social care interventions and inter-sectorial cooperation. Transformation of the current approach to organizing health and social care has been proposed as a way to achieve better continuity in care [1,18]. This, in turn, requires actions on several levels regarding organization and infrastructure along with competence in planning and operating the new forms of integrated services [19,20]. One challenge in any type of transformation of care systems is governance and management [15]. Governing organizational arrangements in which different management structures come together may be challenging [17]. Managers play a significant role in both the implementation and the subsequent management of integrated operations [21]. Managers’ support for a new model has both symbolic and factual significance, in that they are viewed as crucial when organizational changes are introduced [22]. Moreover, leadership is crucial in overcoming the scepticism and protectionism found among professionals regarding collaborative work [6]. To establish and maintain a culture based on collaboration, visionary and stable leadership over a long period of time is needed [23]. Moreover, the leader’s ability to provide appropriate support to professionals in their new roles and teams is of importance [24,25,26]. Leaders’ lack of experience of teams working collaboratively [27] and the occurrence of separate management structures [28] have been suggested as possible reasons, among others, for inefficient management of integrated care. It has been suggested that, to manage integrated services, leaders should be brought together to establish a situation of co-leadership [29]. Recently, the World Health Organization proposed that distributed leadership between multiple actors who work together across professional and organizational boundaries is one key to achieving people-centred and integrated health services [1].

Co-leadership builds on the view that leadership is an activity that several persons can share [30]. The concept of co-leadership was introduced by Heenan and Bennis in 1999 [31]. They defined co-leadership in terms of two leaders equally positioned, sharing the responsibilities of leadership. This conceptualization will serve as a working definition in the present paper. In the literature a variety of concepts can be found, such as collective, shared, collaborative, distributed and emergent leadership [32]. Co-leadership has been shown to have several benefits on the organizational and managerial level, including broader competence and more well-founded decisions [33], personal development and learning, [34], and efficient use of expertise [35]. Barriers to co-leadership addressed in the literature origin in resistance to the model of sharing leadership, as it contradicts the idea that leadership is a singular position [36]. Another limitation concerns the need for more time to reach consensus and take decisions [33]. There are limited amounts of empirical studies on co-leadership, particularly managerial couples sharing the responsibility of management and leadership tasks [37] and even fewer studies on the influence of co-leadership on organizational processes and mechanisms [38]. In general, studies on co-leadership have either been conducted within the health care or the social services sector [39,40,41] alternatively in other sectors as sports, fire brigades, telecom, schools [42] and art [43]. The authors of the present study are not aware of any prior empirical studies that have focused on co-leadership as a managerial solution in integrated health and social care services.

In the present paper, co-leadership exerted in an integrated health and social care service was explored. The aim of the study was to investigate co-leadership in integrated health and social care, identify essential preconditions in fulfilling the management assignment, its operationalization and impact on provision of sustainable integration of health and social care.

The study seeks to answer the following research questions:

What preconditions do managers exerting co-leadership perceive to be essential to fulfil their assignment in an integrated health and social care organization?

How do managers exerting co-leadership operationalize their assignment and how do they perceive its impact on provision of sustainable integration of health and social care?

## Methods

### Setting

The present project was undertaken in an integrated health and social care organization in the region of Stockholm, Sweden. This organization has been in operation since 1995 and was chosen as the object of our study because it has achieved long-lasting, close cooperation and successfully implemented a shared treatment model between mental health services provided by the county council and the municipal social services. The project builds on previous studies of the same case [44,45]. These studies have demonstrated the likely significance of co-leadership for the development and maintenance of integration. In the present study, we focus on four co-located centres belonging to the integrated organization. These centres serve persons over 18 years who have chronic and severe mental illness causing permanent disabilities and who are in need of both mental health and social services. Although managed as one integrated service, the centres are regulated by separate legislation: the Swedish Health and Medical Care Services Act (1982: 763) and the Swedish Social Service Act (2001: 453). The health and social care offered by the centres is organized in interprofessional teams of 10–20 employees from the mental health services provided by county council and the municipal social services. The professional groups mainly include nurses, social workers, physiotherapists, occupational therapists and psychiatrists.

### Study participants and case characteristics

Each centre is managed through co-leadership shared by two equal leaders. The leaders themselves refer to this solution as *pair-leadership* indicating that they in pairs share the responsibility of management. Thus, the study participants are first-line managers having their origin in the two principal organizations. They share responsibility for the mental health and social care services and the management of the teams. Each leader exerts the authority of and is held accountable to either the county council or the municipal part of the service, although they jointly manage the service as one unit. The co-leadership includes responsibility for the budget, work environment issues, human resources, daily operations and development of services. The managers work in close collaboration with the staff and service users by being part of the team. They spend most of their time at the centres and all new service users have assessment interviews with both managers.

Co-leadership is also carried out at the strategic management level, where both principal organizations are represented. Further, the organization is characterized by a collaborative structure in which the municipality and the county council have joined their operations in a model of governance. In addition, a new law (2010: 630) came into force in 2010 requiring cooperation between municipalities and county councils. A shared policy for integrated organization governs overarching operations and activities, while each centre has its local agreements and steering documents, which provide details concerning care and service procedures.

All managers at the centres were included. Thus, a total of eight managers (collaborating in 4 pairs) participated. Managers representing the mental health care organization were educated in nursing, while managers in social care had a background in various social and welfare-related educational programmes. All managers had prior experience of general management positions and the majority of them also of co-leadership. The number of years the managers had collaborated in these pairs ranged from 1–14. Half of the study participants were women and half of them were men.

### Data collection

The data collection method consisted of semi-structured pair interviews conducted in two stages. Prior to the first stage interviews, one researcher (CK) approached the managers by e-mail to inform about the study aim and to obtain informed consent for participation. These interviews took place in October and November 2014 and were conducted by two researchers (CK, JH) at the respondents’ workplaces. The interviews generally lasted 50 minutes and covered questions intended to capture the informants’ perceptions of essential preconditions for co-leadership, its operationalization and impact on provision of sustainable integration of health and social care. The questions were both open ended and targeted to ensure that all possible occurrence of the phenomenon was covered.

The second stage interviews were held during the second stage, the so-called respondent validation [46] were undertaken in order to clarify and elaborate on how our initial findings contributed to sustainable integration of health and social care. A request to participate in the second stage interviews was sent by e-mail to all managers in April 2015; the interviews were held in May 2015. The interviews were carried out on the phone. Only one of the managers in each pair took part in the interview. The interview questions had been sent by email to all the managers in beforehand to enable each pair of managers to have joint discussions before the interviews. The objective was to ask for the managers´ estimation of the extent to which they believed their approach and collective actions contributed to provision of sustainable integration of health and social care. The questions covered management tasks, daily operations and leadership development. One pair of managers was no longer working in the organization due to personal reasons, which is why only six managers were included. These interviews were held over the telephone by the first author and generally lasted 30 minutes per manager.

### Data analysis

All interviews were recorded, transcribed verbatim and analysed using directed content analyses, as described by Hsieh and Shannon [47]. This deductive approach was chosen as it provides a structural process for data gathering and analysis. During the first stage of the analysis, two of the researchers (CK, MAS) read the transcriptions separately to obtain an in-depth understanding of the data. Thereafter all text sections that on first impression seemed to respond to the overarching research questions were highlighted. This stage was followed by coding and subcategorizing made separately by the same researchers. The next steps brought together the subcategories and, in this way, identifying key themes and developing general categories. The categorization helped to indicate how co-leadership and the preconditions were perceived as well as the extent to which it contributed to the provision of sustainable integration of health and social care. To confirm our conclusion, additional data collection in the form of second stage semi-structured interviews was carried out. Finally, the interview data were analysed collectively by the research team (all authors) to arrive at the final categories and themes through a process of negotiated consensus [48]. The quotations in the results were chosen as they in an illustrative manner reflect the informants’ perceptions. The inclusion of equal number of quotes from all informants was ensured.

## Results

This section is divided into two parts, the first of which presents the analysis of the informants’ statements concerning the essential preconditions for exertion of co-leadership. The second part concerns operationalization of co-leadership and its impact on provision of sustainable integration of health and social care.

### Part one: essential preconditions for exertion of co-leadership

Two categories concerning the essential preconditions for exertion of co-leadership were identified: contextual as well as personal and interpersonal preconditions.

#### Contextual preconditions

Among contextual preconditions two subcategories emerged: the overall health and social care organization and co-location of the centres.

Joint efforts to build up the integrated organization over a long period of time and the strongly anchored idea of co-leadership as a management solution played a critical role in the success of co-leadership. The co-leadership on the next managerial level was identified as an important precondition, as the superior managers were perceived as role models and bearers of the culture.

“The fact that this pair-model is so established has helped us. […] You could say that the surrounding context is very good. The whole organization, the psychiatric centre and municipality have built all this up and it really is to our advantage”.

Co-location of each centre was pointed out as an enabling precondition for the management of a common health and social care service. The informants emphasized the importance of co-location for exertion of co-leadership, teamwork as well as for the benefit for the service users.

“Here cooperation is easy, but it’s not always this easy. Cooperating is more difficult the more geographical distance there is. […] Just the fact that we’re in the same building is very important because there’s a lot of informal conversations and contacts. That’s what facilitates things and comes through somehow. Of course it also helps that we have joint meetings and teams and the like, but the simple fact that we see each other during regular work days means that at meetings we can focus on the things we need the whole team to talk about”.

Having one common mission for the whole mental health and social care service guiding the daily work was also emphasized as important. Furthermore creation of one culture that unifies all staff was reported as an important precondition for jointly leading an integrated service.

“It’s really crucial to stress the importance of cooperation, collaboration, in all contexts. That we’re supposed to find a solution that’s best for the client, not mainly discuss whether it’s the county council or the municipality, but what the solution is. And then when we know what the client needs, we find a way to meet those needs. It has to reflect how we act as well as how my staff acts. […] Then you have to understand what the other, well, what the municipality’s and the county council’s duties are. […] And you also have to have a common mission so that you’re not always defending your own side”.

#### Personal and interpersonal preconditions

Among personal and interpersonal preconditions two subcategories emerged: management role and personal characteristics and abilities.

Perceiving the management role as a collective activity and having a common understanding of the purpose of providing integrated health and social care was stated as important. In contact with staff and service users the importance of being clear about the management team consisting of two equal leaders managing one common service was also stressed. Being interested in and willing to invest time in collaboration and in learning about each other’s responsibilities and sector-specific activities was crucial to understanding and managing the big picture.

“The fact that we at least try to learn a bit about each other’s areas of responsibility. You’re almost forced to somehow, for the sake of the whole”.

It was emphasized that getting along with one’s leader-colleague was a key precondition for working together, side-by-side, and for fruitful cooperation. Characteristics such as responsiveness, lack of prestige and self-confidence were highlighted as important. Interaction abilities and transparency were also stressed as crucial. Other important preconditions on the relational level were having the ability to rely on one´s leader-colleague, allowing one to be influenced by him or her as well as being able to compromise. Openness and constant communication including, e.g., sharing information and striving to achieve consensus, were underlined as essential to successful co-leadership. A creation of a trustful and loyal relationship was indispensable, as the confidence that emerged from trust and loyalty provided a space for mistakes to be made without jeopardizing the relationship.

“So I think we have to be loyal to each other. There has to be loyalty, then you’re secure in your joint leadership. There’s a margin for error, I know X will stick by me anyway. Like in a marriage. Exactly”.

### Part two: Co-leadership in practice and its contribution to sustainable integration of health and social care

Three categories related to the daily practice of co-leadership were identified: management tasks, daily operations and leadership development. This section also contains the confirmations from second stage interviews regarding the informants’ assessment of various activities’ degree of contribution to sustainable integration of health and social care.

#### Management tasks

Managing integrated services by exercising co-leadership was characterized by keeping the resources together in terms of staff, which required that both leaders were willing to “give and take” and occasionally even “step back”. This flexibility was described as one key to achieving sustainable integration of health and social care, as stated in the second stage interviews.

“We’ve done a lot to come together here and we’ve seen that it pays off for both of us, so to speak, not to split the resources up internally so they end up somewhere else, but instead to try to keep them together so it becomes a joint responsibility and it’s that ambition that makes it fun to work here. And that’s the point of it all because then you can build a team that’s multi-qualified that masters the whole picture and there’s knowledge about the whole, surrounding the client”.

A common approach to managing services was by involving all team members in the process of finding cross-effective solutions irrespective of where in the integrated mental health and social care organization the problem originated. By involving everyone in the process, the managers attempted to achieve a feeling of solidarity among the team members, which in turn was a major contributing factor to provision of sustainable integration of health and social care, according to the second stage interviews.

“The county council’s administrative side has increased enormously. Then I didn’t understand that it was we and them, but it affects the whole staff group because we see ourselves as a big team, the whole group here. […] Then we worked with, gee, how can the municipal staff help increase the production side here […] If we don’t produce, that means I’ll have to fire people. We have to help each other. And then things got much, much better. This is a major driving force behind cooperation between the municipality and the county council. It’s exciting. It’s always a matter of give and take”.

One way to achieve a common approach to provide equal health and social care irrespective of centre was to hold joint meetings for all managers exercising co-leadership and their superiors. This was described as central in maintaining sustainable integration of health and social care, according to the second stage interviews.

“Then we have meetings where all the managers and physicians are involved as well as our superiors. I must say the group really feels whole, complete. I find it very open; you can say what you want. […] Lots of creative ideas are conceived. […] We take many decisions in the group, and we take a lot of quick decisions here too”.

#### Daily operations

Office sharing was said to be important for keeping abreast of what is happening in the centre. It contributed to natural updates on the service situation as well as involvement in matters concerning both sectors. These informal conversations also helped to make the formal meetings more focused. The importance of sharing office for the achievement of a sustainable integration of health and social care was confirmed in the second stage interviews.

“We’re up-to-date together, you know, and when our staff members come in they see both of us, which gives the impression that we’re both answering them. It’s good I think. That way I’m involved in municipal business that also concerns the county council. It’s really a good thing. […] We’re both involved in each other’s duties”.

Co-leadership offered greater presence of leadership, since it gave the managers the opportunity to cover for each other during vacations and meetings outside the centre. In order to emphasize the teamwork being done by the managers and thereby providing role models for the staff, the managers held performance appraisals and meetings together. The importance of presenting themselves as a united team was highlighted in the second stage interviews as a key factor in achieving sustainable integration of health and social care.

“It’s important that the team members perceive we’re a unit. […] It’s not possible to drive a wedge between us and try to separate us. […] If we have work place meetings, for example, then there has to be two leaders who alternate. So it’s not just one of us on the stage and the other in the background. So you have to constantly be thinking about taking the baton. About passing the baton on to each other”.

As a result of the broad competence the co-leaders achieved by combining their different areas of expertise they perceived themselves as better equipped to manage a cross-boundary service. All service-user-related work was perceived to benefit from the holistic approach that was achieved through different competencies, missions and responsibilities of the managers. This also helped them make faster decisions about health and social care interventions. Thus, service users received faster access to care and support from both the county council and the municipality. The second stage interviews confirmed the importance of the broad managerial competence in achieving sustainable integration of health and social care.

“If nothing else it’s the service users who get this access. Rapid assistance with their needs when we see that the patients aren’t doing well. Right, we have both the county council and the municipality here. The county council with its resources and the municipality with its resources. That’s what it’s all about, benefiting the target group of people living with psychosis, helping them improve their quality of life. […] Everything happens here at the centre and it happens quickly”.

#### Leadership development

Joint decision-making was described as something that could be challenging, especially if one´s previous experience was being a single manager. However, the difficulties related to acting as part of a co-leadership team are outweighed by the advantages, in terms of self-development and the sense of confidence which derives from never being alone. Co-leadership was also said to provide the advantage of immediate guidance and mentoring from one’s leader colleague. The support they gave one another was described as resulting in more robust management and, thus, as contributing to provision of sustainable, health and social care.

“Actually, it’s easier to lead alone than to be two leaders. You have to wait all the time, but still it’s more fulfilling because I have someone to share things with. It’s more difficult but you get more out of it and you have to work more on yourself.”

“If I can get it out immediately, if I can shut the door, put the red light on and tell X […] then I can verbalize things, which you can’t do when you’re alone […] then I’ve got it off my chest. I can go home, I don’t have to go around thinking how I should express myself and do something about the situation. It’s enough, it’s a kind of direct guidance.”

## Discussion

One of the most significant findings in the present study was the importance of co-leadership for achieving sustainable integration of health and social care. By the advantage of being two managers with different knowledge and responsibilities, the managers could complement each other’s areas of expertise. This finding confirms the results of previous research [33,49,50,51]. Co-leadership exerted in an integrated and co-located centre allowed the managers to deal with service users’ needs and problems in a more holistic and efficient way. Another advantage of co-leadership was thanks to the continuous cooperation, the creation of an environment for managers’ learning and support.

The organization-wide model of cross-boundary co-operation, which recognized co-leadership as a managerial solution, was an important precondition for performance of co-leadership in integrated health and social care. In line with previous research [52,53], having adequate organizational support, i.e. policy strategies enabling co-leadership as a management solution throughout the organization, and having clear common objectives for the services were emphasized as key pre-conditions. The exertion of co-leadership in organizations with less supportive overall policy and administrative structure might thus be challenging. Another important precondition, seen as essential to achieving efficient joint governance of an integrated health and social care service, was co-location. The results of this study are in line with previous findings [16,24,54] showing that co-location enables informal discussions, sharing of knowledge and experience as well as smooth information transfer. Additional essential preconditions for exertion of co-leadership were related to understanding the value of managing care jointly and to viewing management as a collective activity. Other authors have also raised the issue of co-leadership [17] and of having a common understanding of the objectives and visions for the services [13,17] in order to overcome the challenges of cross-boundary integration. The relationship between the managers was characterized by openness, lack of prestige and the managers’ willingness to take a step back, thus allowing their colleague to take the foreground. The results of the present study are consistent with previous findings emphasizing the importance of personal relationships between managers in different organizations when providing integrated care [15,33,34,35].

One practical implication of the findings is that integrated services should consider co-leadership as a possible managerial solution. It is reasonable to assume that the challenges managers are facing in integrated care settings can be handled more efficiently by two managers working together than by a single manager acting alone. However, more research on this topic is needed to establish the association between co-leadership and provision of sustainable integration of health and social care. Future research efforts within the field of co-leadership in various forms of integrated organizations are encouraged. This is an important task, given the challenges faced by today’s managers working in the complex area of health and social care. No previous studies on co-leadership in cross-boundary cooperative settings as integrated health and social care were found. Thus the present study adds one part to a picture that needs to be further developed.

So, what can be learned from this study? One lesson is the advantage of being two managers with different areas of responsibilities working together in integrated health and social care services as it enabled a provision of a more holistic care. However, our findings may apply to a wider setting than integrated care services by addressing co-leadership as a managerial solution to meet managerial challenges in complex organizations. In this study the complex organization was an cross-boundary cooperation between county council mental health service and a municipal social service but the organizational complexity are found in other systems as well. Another lesson that can be transferred to other contexts is the opportunity of support, learning and broader competence that is given by being two managers. These findings have theoretical implications as it provide us with deeper understanding about the theoretical usefulness of co-leadership, its essential prerequisites, operationalization and impact of provision of organizational maintenance.

### Methodological discussion

The present study collected data from one cross-boundary cooperative setting, an integrated health and social care service. The current integration and the model of co-leadership had been in place for 20 years. Thus, the findings from this specific case illustrate important information of essential preconditions for the exercise of co-leadership, its operationalization and impact on sustainability i.e. long term maintenance of integrated care. These findings might not apply to newly established integrated services. Thus, further studies within this field are needed. Furthermore, the findings are primarily relevant to cross-boundary integration. However, the in-depth interviews captured a consistent description of the managers own perceptions of exerting co-leadership. Some of the findings might nevertheless apply to other type of integrated services and provide valuable insights into co-leadership as a managerial solution.

## Conclusion

The present study extends our knowledge of co-leadership as a way of addressing managerial challenges in cross-boundary services as integrated health and social care organizations. Co-leadership enabled robust management by providing broader competence, continuous learning and joint responsibility for services. Therefore, co-leadership can be said to contribute to provision of sustainable integration of health and social care. Essential contextual preconditions for successful co-leadership are having an organization-wide model that supports such management as well as co-location of services. On the personal and interpersonal level, the prerequisites are perception of the management role as a collective activity, continuous communication and lack of prestige. Finally, integrated services aiming to achieve sustainability in integrated health and social care should consider co-leadership as a managerial solution.
